# Erratum: Qin, W., *et al.* Light Cigarette Smoking Increases Risk of All-Cause and Cause-Specific Mortality: Findings from the NHIS Cohort Study. *Int. J. Environ Res. Public Health. 2020, 17, E5122*

**DOI:** 10.3390/ijerph17176176

**Published:** 2020-08-25

**Authors:** Wen Qin, Costan G Magnussen, Shengxu Li, Lyn M Steffen, Bo Xi, Min Zhao

**Affiliations:** 1 Shandong University Hospital, Cheeloo College of Medicine, Shandong University, Jinan 250012, China; qinwen@sdu.edu.cn; 2 Menzies Institute for Medical Research, University of Tasmania, Hobart 7000, Australia; costan.magnussen@utas.edu.au; 3 Research Centre of Applied and Preventive Cardiovascular Medicine, University of Turku, 20520 Turku, Finland; 4 Children’s Minnesota Research Institute, Children’s Hospitals and Clinics, Minneapolis, MN 55404, USA; sli10@tulane.edu; 5 Division of Epidemiology and Community Health, School of Public Health, University of Minnesota, Minneapolis, MN 55454, USA; steff025@umn.edu; 6 Department of Epidemiology, School of Public Health, Cheeloo College of Medicine, Shandong University, Jinan 250012, China; xibo2007@126.com; 7 Department of Nutrition and Food Hygiene, School of Public Health, Cheeloo College of Medicine, Shandong University, Jinan 250012, China

The authors wish to correct the following erratum in this paper [1].

The contributions for the second author (Costan G Magnussen) and the fifth author (Bo Xi) were not fully stated in “Author Contributions” section. Both authors have made substantial contributions to analysis and interpretation of data for the work; AND revising the work critically for important intellectual content; AND final approval of the version to be published; AND agreement to be accountable for all aspects of the work in ensuring that questions related to the accuracy or integrity of any part of the work are appropriately investigated and resolved.
