# Supplementary material for: Light Cigarette Smoking Increases Risk of All-Cause and Cause-Specific Mortality: Findings from the NHIS Cohort Study
Source: Int J Environ Res Public Health. 2020 Jul 15;17(14):5122. doi: 10.3390/ijerph17145122 (PMC7399798; doi:10.3390/ijerph17145122)

Supplemental files

Table S1. Meta-analyses of cigarette smoking status with risk of all-cause mortality according to baseline survey year

**Survey year**	**Smoking status, HRs (95% CIs)**							
			**Current smoker**, cigarettes per day					
	**Never**	**Former smoker**	1-2	3-5	6-10	11-20	21-30	>30
1997	1	1.27 (1.18-1.37)	2.05 (1.63-2.57)	1.68 (1.34-2.09)	2.08 (1.77-2.44)	2.48 (2.19-2.80)	2.32 (1.90-2.83)	3.45 (2.87-4.15)
1998	1	1.39 (1.28-1.51)	1.60 (1.22-2.11)	1.95 (1.57-2.43)	2.20 (1.85-2.62)	2.35 (2.07-2.68)	2.86 (2.31-3.53)	3.35 (2.72-4.12)
1999	1	1.29 (1.18-1.40)	1.83 (1.33-2.50)	1.93 (1.46-2.54)	2.33 (1.97-2.76)	2.38 (2.07-2.72)	2.58 (2.03-3.29)	3.57 (2.79-4.58)
2000	1	1.44 (1.32-1.57)	1.91 (1.43-2.57)	2.35 (1.81-3.04)	2.03 (1.68-2.46)	2.01 (1.72-2.35)	3.35 (2.62-4.29)	3.60 (2.78-4.66)
2001	1	1.29 (1.18-1.41)	1.94 (1.47-2.56)	1.88 (1.45-2.44)	1.82 (1.48-2.23)	2.28 (1.95-2.67)	2.25 (1.65-3.07)	3.29 (2.54-4.28)
2002	1	1.21 (1.09-1.35)	1.26 (0.78-2.02)	2.07 (1.58-2.71)	2.32 (1.91-2.81)	2.16 (1.82-2.57)	2.40 (1.70-3.39)	3.64 (2.83-4.69)
2003	1	1.20 (1.08-1.34)	1.67 (1.11-2.51)	1.88 (1.41-2.50)	1.85 (1.46-2.36)	2.08 (1.72-2.52)	2.06 (1.40-3.02)	3.63 (2.59-5.07)
2004	1	1.29 (1.15-1.44)	2.53 (1.78-3.61)	2.27 (1.53-3.37)	1.58 (1.24-2.02)	1.98 (1.60-2.44)	3.23 (2.37-4.41)	2.12 (1.37-3.30)
2005	1	1.40 (1.24-1.58)	1.77 (1.11-2.80)	1.89 (1.31-2.72)	1.76 (1.36-2.27)	2.44 (1.97-3.02)	2.89 (1.91-4.38)	3.81 (2.56-5.68)
2006	1	1.53 (1.31-1.78)	1.92 (1.04-3.55)	2.36 (1.54-3.62)	1.90 (1.35-2.67)	2.09 (1.60-2.74)	3.73 (2.41-5.79)	2.34 (1.48-3.70)
2007	1	1.55 (1.27-1.88)	2.48 (1.58-3.89)	1.85 (1.01-3.41)	2.45 (1.64-3.65)	1.99 (1.38-2.89)	1.43 (0.75-2.74)	3.30 (1.79-6.07)
2008	1	1.42 (1.15-1.75)	3.02 (1.49-6.12)	1.91 (0.97-3.77)	2.94 (1.92-4.51)	2.16 (1.50-3.10)	3.78 (2.11-6.78)	4.03 (2.38-6.84)
2009	1	1.49 (1.14-1.95)	3.27 (1.68-6.38)	2.28 (1.24-4.18)	1.42 (0.90-2.22)	1.69 (1.07-2.67)	2.10 (0.84-5.26)	2.73 (1.00-7.44)
I^2 (%)		42.3	9.6	0	34.9	0	33.1	0
Statistical Model		Fixed-effects	Fixed-effects	Fixed-effects	Fixed-effects	Fixed-effects	Fixed-effects	Fixed-effects
Meta-analyses	1	1.33 (1.29-1.37)	1.94 (1.75-2.14)	1.97 (1.81-2.15)	2.05 (1.93-2.18)	2.25 (2.14-2.36)	2.67 (2.45-2.91)	3.39 (3.12-3.69)

Cox proportional hazards regression models were adjusted for sex, age, race/ethnicity, education, marital status, body mass index, alcohol intake, physical activity and physician-diagnosed diseases (hypertension, heart disease, stroke, cancer and diabetes)

Table S2. Cigarette smoking status and risk of all-cause and cause-specific mortality (Exclusion of participants who died within the first 2 years of follow up)

	Smoking status, HRs (95% CIs)							
			**Current smoker**, cigarettes per day					
	**Never**	**Former smoker**	1-2	3-5	6-10	11-20	21-30	>30
**All causes**	1	1.31 (1.26-1.35)	1.75 (1.55-1.97)	1.98 (1.80-2.17)	2.01 (1.87-2.16)	2.32 (2.20-2.45)	2.69 (2.44-2.98)	3.55 (3.24-3.88)
**Cancer**	1	1.60 (1.49-1.72)	2.13 (1.66-2.74)	2.68 (2.21-3.24)	2.37 (2.07-2.71)	3.49 (3.17-3.84)	4.13 (3.51-4.86)	5.66 (4.84-6.62)
**CVD**	1	1.16 (1.09-1.23)	1.92 (1.52-2.41)	2.01 (1.64-2.45)	2.37 (2.01-2.79)	2.44 (2.16-2.74)	2.98 (2.42-3.65)	3.66 (3.01-4.44)
**Heart disease**	1	1.18 (1.10-1.28)	1.80 (1.37-2.36)	1.96 (1.58-2.44)	2.55 (2.10-3.10)	2.64 (2.31-3.02)	3.31 (2.65-4.14)	4.01 (3.27-4.92)
**Cerebrovascular disease**	1	1.10 (0.96-1.25)	2.50 (1.54-4.07)	2.21 (1.42-3.45)	1.93 (1.50-2.50)	1.74 (1.35-2.25)	1.82 (1.17-2.84)	2.41 (1.42-4.09)
**Respiratory diseases**	1	6.93 (5.78-8.31)	7.75 (4.31-13.93)	11.73(8.57-16.07)	13.43(10.53-17.12)	15.29(12.27-19.05)	19.49(13.93-27.28)	30.23(22.39-40.83)

Cox proportional hazards regression models were adjusted for sex, age, race/ethnicity, education, marital status, body mass index, alcohol intake, physical activity and physician-diagnosed diseases (hypertension, heart disease, stroke, cancer and diabetes)

Table S3. Cigarette smoking status and risk of all-cause and cause-specific mortality (Exclusion of participants who had history of physician-diagnosed diseases)

	**Smoking status, HRs (95% CIs)**							
			**Current smoker**, cigarettes per day					
	**Never**	**Former smoker**	1-2	3-5	6-10	11-20	21-30	>30
**All causes**	1	1.29 (1.21-1.37)	1.71 (1.40-2.09)	1.99 (1.72-2.31)	1.95 (1.77-2.15)	2.25 (2.07-2.45)	2.56 (2.17-3.03)	3.69 (3.21-4.25)
**Cancer**	1	1.60 (1.41-1.81)	1.67 (1.11-2.53)	2.73 (2.00-3.74)	2.08 (1.70-2.54)	3.39 (2.91-3.95)	4.44 (3.46-5.69)	6.30 (5.00-7.95)
**CVD**	1	1.04 (0.90-1.19)	1.93 (1.23-3.04)	1.77 (1.24-2.52)	2.21 (1.75-2.81)	2.91 (2.42-3.51)	2.96 (2.15-4.08)	3.79 (2.67-5.37)
**Heart disease**	1	1.10 (0.94-1.28)	2.32 (1.42-3.81)	1.79 (1.19-2.69)	2.20 (1.64-2.95)	3.11 (2.53-3.82)	3.51 (2.49-4.94)	4.35 (2.98-6.34)
**Respiratory diseases**	1	7.13 (5.28-9.64)	5.97 (2.36-15.06)	9.77 (5.85-16.31)	11.02(7.22-16.83)	13.82(9.64-19.82)	15.05(8.43-26.84)	31.12(19.12-50.66)

Cox proportional hazards regression models were adjusted for sex, age, race/ethnicity, education, marital status, body mass index, alcohol intake, and physical activity

Note: HRs (95% CIs) for cerebrovascular disease were not calculated because of limited number of deaths

Table S4. Cigarette smoking status and risk of all-cause and cause-specific mortality (using imputed data)

	Smoking status, HRs (95% CIs)							
			**Current smoker**, cigarettes per day					
	**Never**	**Former smoker**	1-2	3-5	6-10	11-20	21-30	>30
**All causes**	1	1.32 (1.28-1.36)	1.96 (1.76-2.18)	1.97 (1.82-2.14)	2.02 (1.90-2.14)	2.18 (2.08-2.29)	2.59 (2.38-2.83)	3.16 (2.91-3.43)
**Cancer**	1	1.64 (1.55-1.74)	2.24 (1.82-2.76)	2.64 (2.23-3.12)	2.47 (2.21-2.75)	3.22 (2.96-3.51)	4.03 (3.51-4.64)	4.94 (4.27-5.71)
**CVD**	1	1.18 (1.11-1.25)	2.04 (1.67-2.50)	1.92 (1.61-2.28)	2.25 (1.97-2.57)	2.27 (2.04-2.53)	3.04 (2.55-3.63)	3.32 (2.79-3.94)
**Heart disease**	1	1.22 (1.14-1.30)	2.03 (1.59-2.59)	1.86 (1.55-2.24)	2.37 (2.03-2.77)	2.39 (2.12-2.70)	3.43 (2.83-4.15)	3.59 (2.99-4.32)
**Cerebrovascular disease**	1	1.06 (0.94-1.20)	2.39 (1.58-3.63)	2.19 (1.48-3.25)	1.91 (1.50-2.44)	1.83 (1.46-2.29)	1.68 (1.10-2.56)	2.25 (1.44-3.53)
**Respiratory diseases**	1	6.63 (5.64-7.81)	9.05 (5.65-14.49)	12.13(9.33-15.78)	12.67(10.12-15.85)	13.65 (11.21-16.60)	17.25(12.70-23.43)	27.30(20.48-36.38)

Cox proportional hazards regression models were adjusted for sex, age, race/ethnicity, education, marital status, body mass index, alcohol intake, physical activity and physician-diagnosed diseases (hypertension, heart disease, stroke, cancer and diabetes)

Table S5. Cigarette smoking status and risk of all-cause mortality by sex, age group and race/ethnicity

	Smoking status, HRs (95% CIs)							
			**Current smoker**, cigarettes per day					
	**Never**	**Former smoker**	1-2	3-5	6-10	11-20	21-30	>30
**Sex**								
Men	1	1.28 (1.22-1.34)	1.73 (1.48-2.03)	1.98 (1.73-2.26)	2.00 (1.83-2.18)	2.14 (1.99-2.29)	2.49 (2.22-2.79)	2.99 (2.69-3.33)
Women	1	1.42 (1.36-1.48)	2.18 (1.87-2.53)	2.00 (1.76-2.26)	2.10 (1.93-2.29)	2.30 (2.15-2.47)	2.78 (2.42-3.20)	4.05 (3.43-4.78)
**Age group, years**								
18-39	1	1.17 (0.96-1.41)	1.66 (1.15-2.40)	1.54 (1.14-2.06)	1.66 (1.35-2.04)	1.65 (1.37-1.98)	1.81 (1.30-2.51)	2.75 (1.96-3.85)
40-59	1	1.27 (1.17-1.38)	1.69 (1.39-2.04)	1.92 (1.61-2.30)	2.01 (1.77-2.28)	2.45 (2.24-2.68)	2.87 (2.51-3.27)	3.43 (3.02-3.89)
≥60	1	1.40 (1.35-1.45)	2.11 (1.84-2.41)	2.13 (1.90-2.38)	2.12 (1.96-2.29)	2.23 (2.09-2.38)	2.70 (2.37-3.08)	3.26 (2.86-3.71)
**Race/ethnicity**								
White	1	1.38 (1.33-1.43)	2.04 (1.75-2.37)	2.21 (1.97-2.47)	2.13 (1.97-2.29)	2.38 (2.25-2.51)	2.86 (2.59-3.15)	3.57 (3.26-3.91)
Black	1	1.15 (1.05-1.25)	1.88 (1.54-2.29)	1.49 (1.28-1.75)	1.72 (1.52-1.95)	1.80 (1.58-2.05)	1.77 (1.22-2.56)	2.56 (1.90-3.47)
Hispanic	1	1.22 (1.08-1.37)	1.46 (1.14-1.86)	1.56 (1.18-2.06)	1.83 (1.50-2.25)	1.78 (1.47-2.17)	1.36 (0.74-2.49)	3.62 (2.31-5.68)
Other	1	1.05 (0.84-1.32)	1.61 (0.93-2.81)	1.99 (1.26-3.14)	1.48 (0.99-2.23)	1.45 (1.03-2.04)	2.70 (1.41-5.17)	2.30 (1.14-4.65)

Cox proportional hazards regression models were adjusted for sex, age, race/ethnicity, education, marital status, body mass index, alcohol intake, physical activity and physician-diagnosed diseases (hypertension, heart disease, stroke, cancer and diabetes)

Figure S1 Dose-response associations of cigarette smoking with (A) all-cause, (B) cancer-specific mortality, (C) CVD-specific mortality, and (D) respiratory disease-specific mortality in U.S. adults

Figure S1-A: All-cause mortality

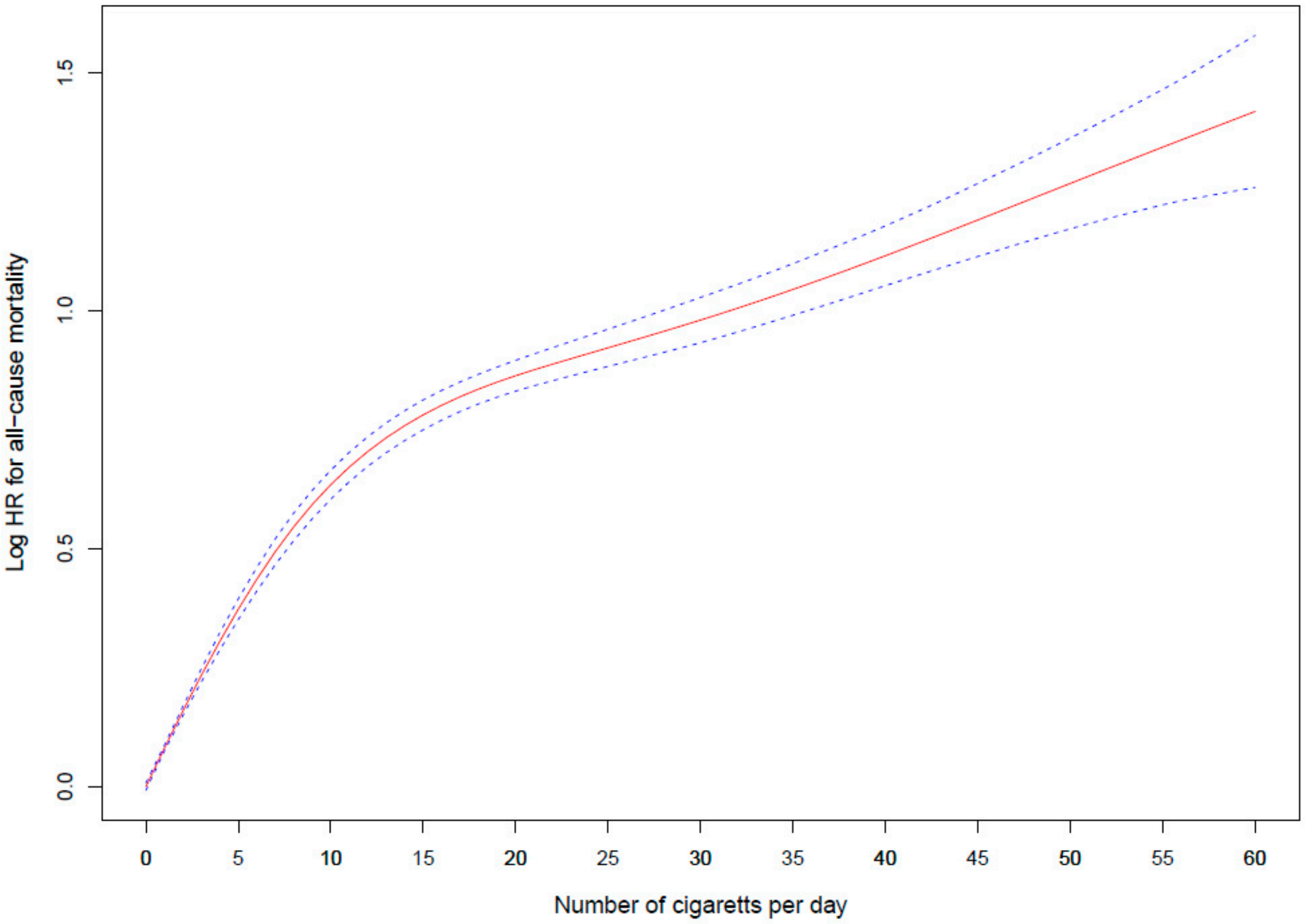

Figure S1-B: Cancer-specific mortality

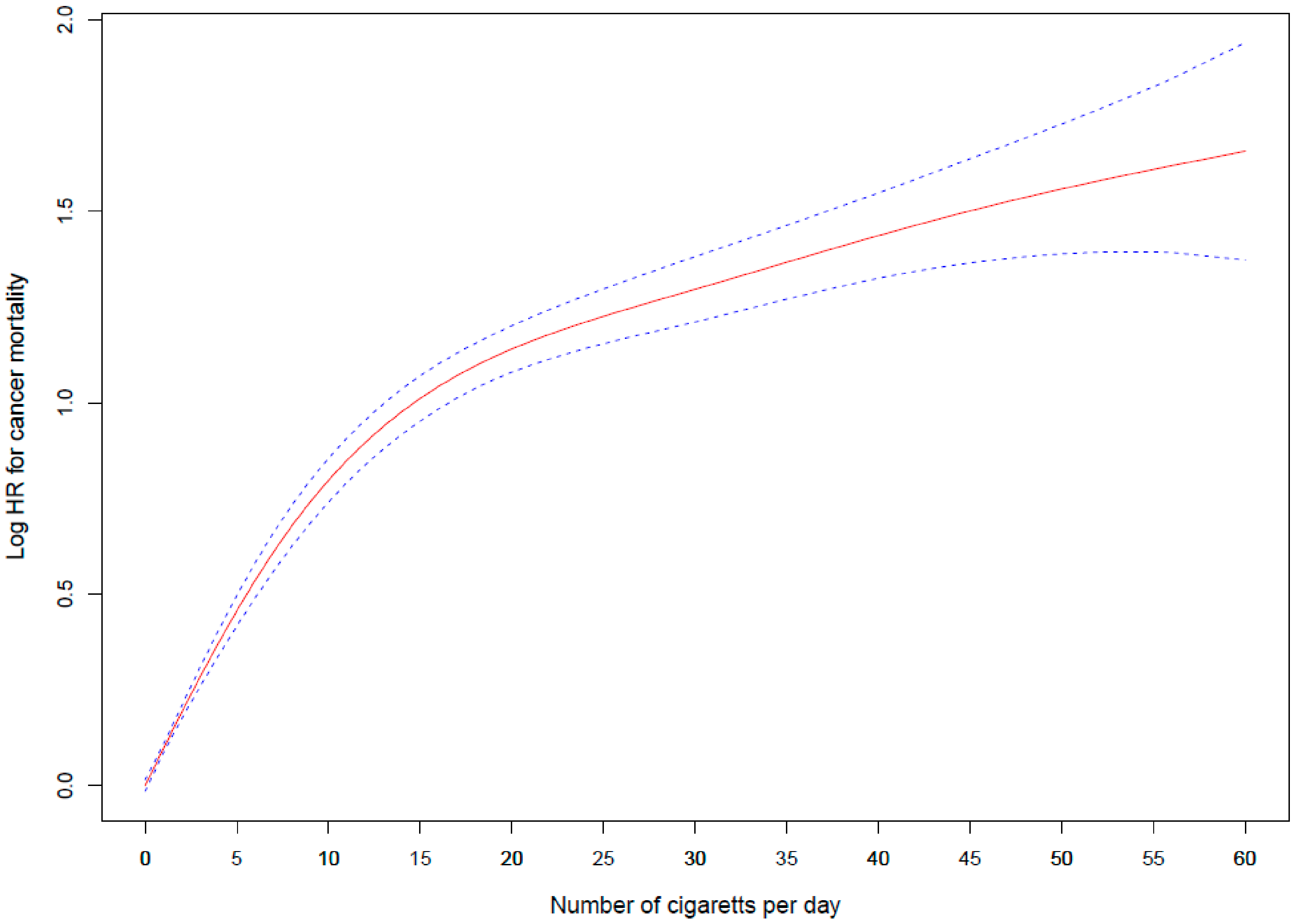

Figure S1-C: CVD-specific mortality

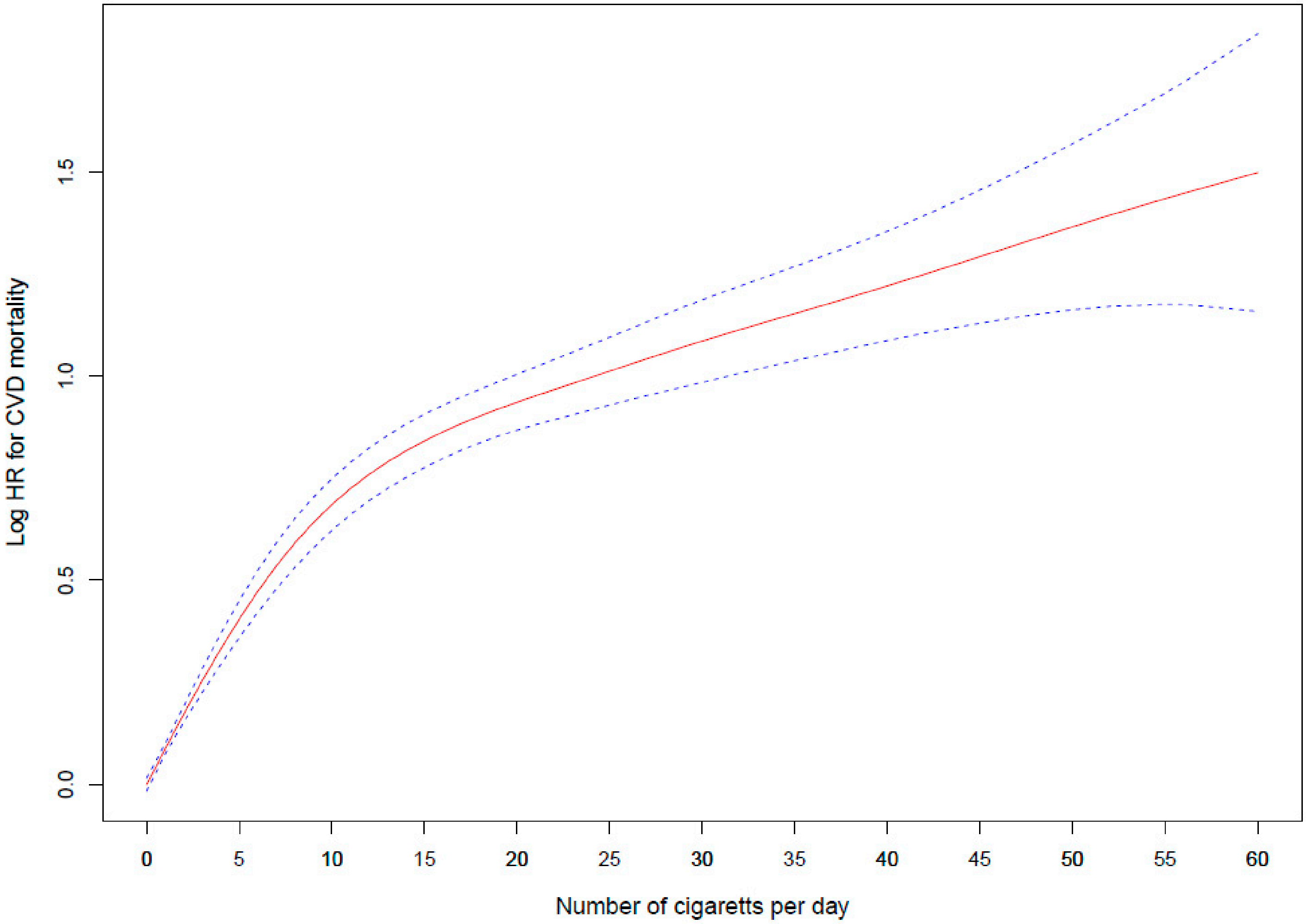

Figure S1D: Respiratory disease-specific mortality

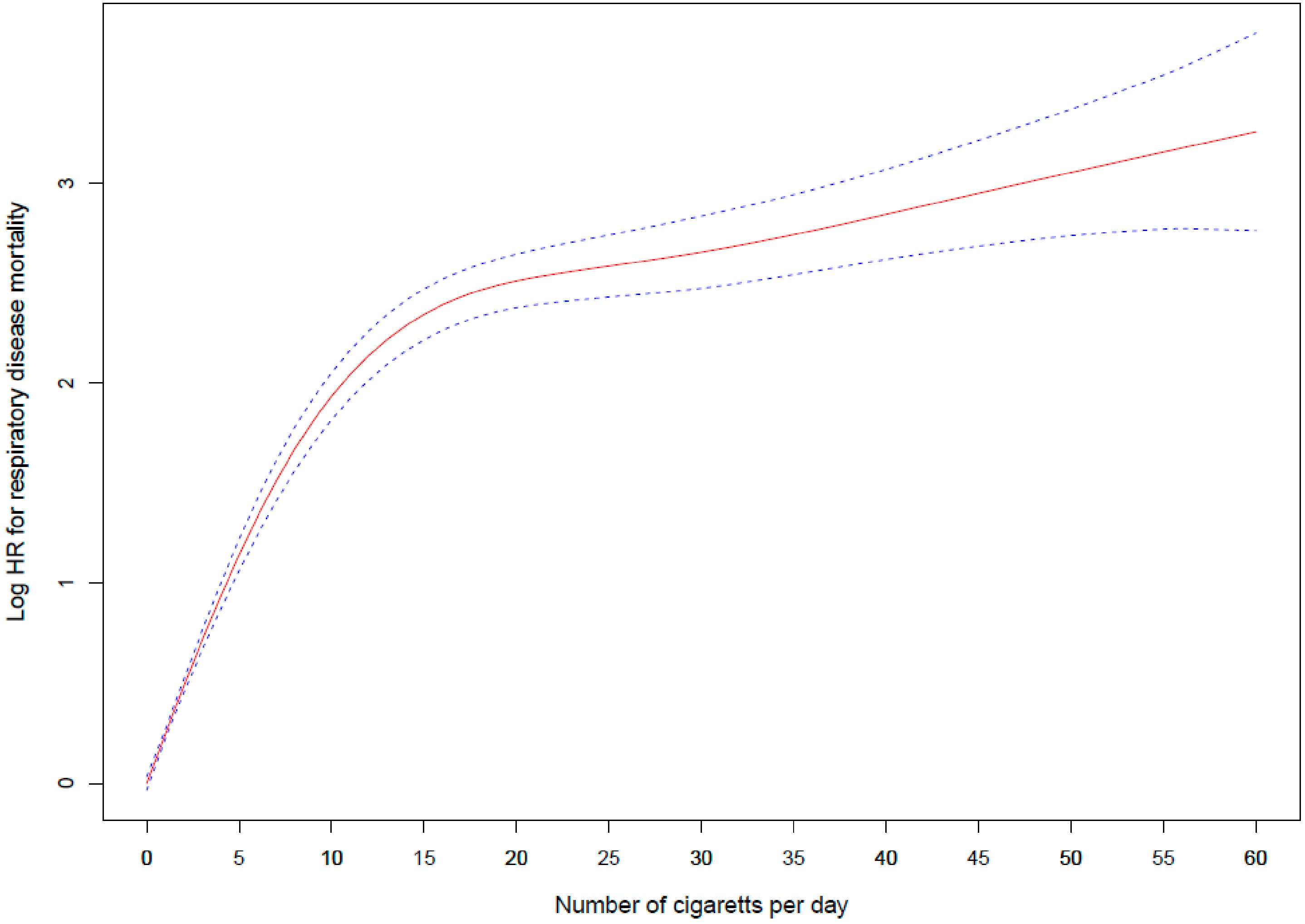

Supplement: Supplementary file 1 [file ijerph-17-05122-s001.pdf]
